# Internet media use, physical activity participation, and health-related outcomes among Chinese adults: evidence from CGSS2023

**DOI:** 10.3389/fpubh.2026.1873007

**Published:** 2026-06-15

**Authors:** Yi Lin, Wenfeng Zhu, Banghui Hong

**Affiliations:** 1 College of Physical Education and Sport Science, Fujian Normal University, Fuzhou, China; 2 School of Physical Education, Guizhou Normal University, Guiyang, China

**Keywords:** Chinese General Social Survey, cross-sectional study, digital lifestyle, health-related outcomes, internet media use, physical activity participation

## Abstract

**Objective:**

Using data from the 2023 Chinese General Social Survey (CGSS2023), this study examined the cross-sectional association between internet media-use frequency and physical activity participation frequency among Chinese adults. It further assessed social-group heterogeneity and evaluated secondary associations with health-related outcomes.

**Methods:**

This cross-sectional observational study included a complete-case analytical sample of 5,621 adults. Multivariable ordinary least squares (OLS) regression was used as the primary descriptive model. Weighted OLS, ordered logit, binary logit and inverse-probability-weighted sensitivity analyses were used to assess robustness. Group heterogeneity was assessed using interaction models.

**Results:**

Internet media-use frequency was positively but modestly associated with physical activity participation frequency in the primary model (b = 0.084, 95% CI 0.049 to 0.119). The estimate remained similar after inverse-probability weighting (b = 0.094, 95% CI 0.058 to 0.130). Ordered-logit marginal effects indicated that a one-level increase in internet media use was associated with a 1.8-percentage-point lower probability of being in the lowest physical-activity category and a 1.1-percentage-point higher probability of being in the highest category. The association was stronger among low-income respondents. Additional lifestyle-overlap variables were statistically related to both internet use and physical activity, and internet use and physical activity each showed small positive associations with self-rated health, mental wellbeing and daily functioning.

**Conclusion:**

Among Chinese adults, more frequent internet media use was associated with slightly more frequent physical activity participation, with the clearest subgroup difference observed by income. These findings support a bounded cross-sectional digital-lifestyle interpretation rather than a causal account of internet use, physical activity or health.

## 1 Introduction

Internet media use and physical activity participation are both increasingly embedded in everyday life, yet their relationship is not theoretically straightforward. Digital media may support exercise by improving access to information, examples and social connection, but frequent online activity may also reflect screen-based leisure and time competition. The key question is therefore not simply whether internet use matters, but how internet media-use frequency is associated with physical activity participation in contemporary adult life (1–3).

Physical activity participation remains a public-health concern in China. National surveillance shows that participation has become socially visible, while regular participation and access to opportunities still vary across population groups. This makes it important to examine how physical activity is patterned within the broader conditions of adult daily life (4).

Physical activity also has clear health relevance. Existing guidelines and empirical studies link physical activity with health-related outcomes such as depression risk, functional status and self-rated health. In the present study, these outcomes are treated as secondary extensions of the behavioral question rather than as evidence of a causal sequence (5–10).

The present study addresses a more specific gap than a simple data update. Existing evidence remains mixed on whether internet use is associated with more or less physical activity, and prior work has often focused on particular age groups or earlier survey waves. Adults are theoretically important because work, family responsibilities, income, education and access to offline sport resources jointly shape both digital participation and leisure allocation. We therefore use a bounded digital-lifestyle perspective in which internet use, offline activity, social participation and health-related outcomes may be statistically co-patterned without assuming that all favorable behaviors belong to a single undifferentiated lifestyle.

**Figure 1:**
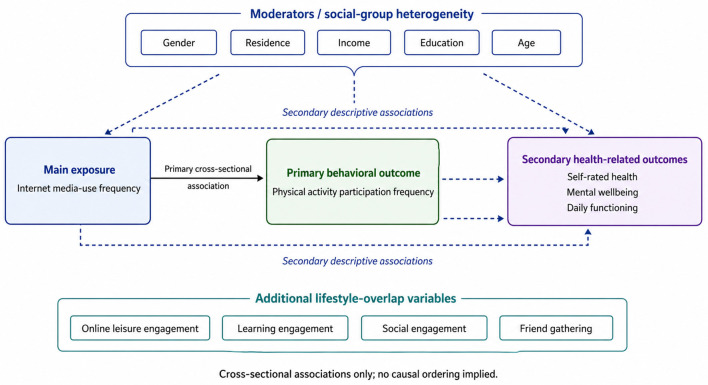
Conceptual framework of the study.

Accordingly, this study uses CGSS2023 data (11) to examine four linked but distinct questions among Chinese adults: the primary association between internet media-use frequency and physical activity participation frequency; whether this association differs across social groups; whether the focal association changes after adding additional lifestyle-overlap variables; and whether internet use and physical activity participation are each associated with health-related outcomes. The analytical framework is summarized in Figure 1.

## 2 Literature review, theoretical framework and hypotheses

Prior studies suggest that internet use may be related to physical activity participation, but they do not resolve the direction or meaning of that association. Digital platforms can expand access to sport information, exercise content and online communities, and studies of Chinese residents and age-specific samples have reported positive associations between internet use and physical activity participation (12–15).

One theoretical explanation emphasizes enabling resources. Social cognitive theory highlights observational learning, self-efficacy, feedback and social environment. In digital settings, exercise videos, online courses, activity check-ins and community interaction may provide information and encouragement that make physical activity easier to organize or sustain (16).

A competing explanation emphasizes sedentary routines and displacement. Internet use may also involve short videos, gaming, entertainment and passive social networking, which can compete with offline exercise or cluster with less active leisure patterns. Prior evidence has therefore also linked internet use with lower physical activity or more sedentary behavior in some settings (17–20).

These competing expectations indicate that internet use is not a single exposure with a uniform behavioral meaning. Internet-based interventions and mobile applications can facilitate activity under some conditions, but their implications depend on the purpose and pattern of use. Because the CGSS2023 item measures overall frequency rather than content-specific use, the present study cannot distinguish exercise-oriented from entertainment-oriented internet use (21–23).

The association may also vary across social groups. Research on the digital divide shows that inequalities extend beyond access to skills, uses and returns from digital participation. These inequalities are intertwined with broader economic, cultural and social resources (24–27).

Physical activity participation is likewise socially patterned by age, gender, health status, social environment and built environment. For adults, income, education, residence and time constraints may shape both digital participation and opportunities for physical activity. Heterogeneity analysis is therefore theoretically relevant rather than merely a routine extension (28–31).

Internet media use and physical activity participation may also overlap statistically with other aspects of everyday life. Online leisure engagement, learning engagement, social engagement and friend gathering capture adjacent activities that may correlate with both digital participation and offline exercise (32–37).

These variables are not treated here as causal mediators, mechanisms or pathways. Strict mediation analysis requires temporal ordering and strong control of confounding, neither of which can be established with the present cross-sectional data. They are therefore examined only as additional lifestyle-overlap variables (38).

Health-related outcomes provide a secondary extension of the framework. Prior research has linked physical activity and internet use with self-rated health, mental wellbeing and related outcomes, but cross-sectional data cannot establish whether internet use, physical activity or health comes first (7–10, 39, 40).

Accordingly, the health analyses in this study are interpreted as descriptive cross-sectional associations that broaden the public-health relevance of the primary behavioral question without implying causal ordering.

Based on this literature review and theoretical framework, this study proposes the following hypotheses:

H1: Higher internet media-use frequency is expected to be associated with more frequent physical activity participation among Chinese adults.

H2: Given socioeconomic differences in digital returns and offline sport resources, the association between internet media-use frequency and physical activity participation frequency is expected to be stronger among socioeconomically constrained groups, particularly low-income respondents; heterogeneity by gender, residence, education and age is examined exploratorily.

H3: The focal association is expected to attenuate after additional lifestyle-overlap variables are added, reflecting shared cross-sectional patterning rather than mediation.

H4: Internet media-use frequency and physical activity participation frequency are each expected to be positively associated with favorable health-related outcomes, while these relationships are interpreted as cross-sectional associations rather than causal effects.

## 3 Materials and methods

### 3.1 Data source and study design

This study used data from the 2023 Chinese General Social Survey (CGSS2023), a nationally representative cross-sectional survey of Chinese residents.

All variables were drawn from the same survey wave. The study was designed to estimate cross-sectional statistical associations rather than causal effects.

The reporting of the Methods section was guided by the Strengthening the Reporting of Observational Studies in Epidemiology (STROBE) recommendations for observational studies. STROBE recommends that cross-sectional studies clearly report data sources, study participants, variable definitions, sample selection, potential sources of bias and statistical methods. Accordingly, this study reports the sample selection process, variable construction, model specification and sensitivity analyses (41).

### 3.2 Study participants and sample selection

**Figure 2:**
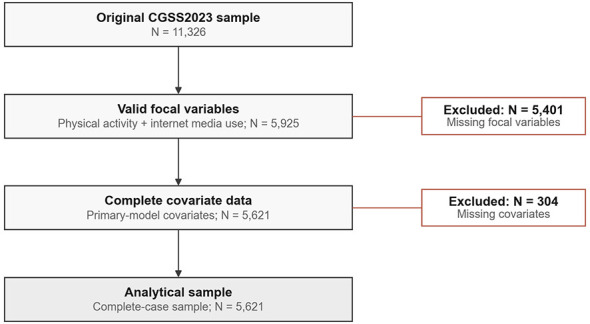
Sample flow diagram.

The original CGSS2023 sample included 11,326 respondents. We first retained respondents with valid information on both physical activity participation frequency and internet media-use frequency, yielding 5,925 observations. The primary analytical sample was then restricted to respondents with non-missing data on all covariates used in the main physical-activity model, yielding a final complete-case sample of 5,621 adults. The sample-selection process is shown in Figure 2.

Complete-case analysis was retained as the primary strategy, but sample selection was treated as a substantive methodological issue. We report variable-specific missingness, sample-flow counts, comparisons between included and excluded respondents, and an inverse-probability-weighted sensitivity analysis based on observed predictors of inclusion in the analytical sample. IPW was selected as the missing-data sensitivity strategy because it directly evaluates whether the main estimate is stable after reweighting complete cases to resemble the observed CGSS2023 sample on measured predictors of analytical-sample inclusion. Multiple imputation was not used because the main missingness was concentrated in the ordinal focal behavioral variables and lifestyle-overlap variables, and imputing these variables would require stronger missing-at-random and ordinal imputation-model assumptions. Complete-case analysis and reweighting were used with explicit recognition that missing-data assumptions can affect epidemiological estimates (42).

### 3.3 Variable measurement

#### 3.3.1 Physical activity participation frequency

The main dependent variable was physical activity participation frequency, measured by CGSS2023 item a3009. The original response categories ranged from every day to never and were reverse-coded so that 1 = never and 5 = every day. The distribution is shown in Supplementary Figure S1.

1 = never, 2 = several times a year or less, 3 = several times a month, 4 = several times a week and 5 = every day.

Higher values indicate more frequent participation in physical activity.

For robustness analyses, a binary frequency-based physical activity variable was also constructed. Respondents reporting physical activity every day or several times a week were coded as 1, whereas those reporting several times a month or less often were coded as 0. This cutoff was used only as a pragmatic frequency-based robustness measure and should not be interpreted as compliance with WHO physical-activity guidelines because the survey item does not measure duration or intensity.

#### 3.3.2 Internet media use frequency

The key explanatory variable was internet media-use frequency, measured by CGSS2023 item a285. The variable ranged from 1 to 5, with higher values indicating more frequent internet media use. Because the item captures overall frequency only, it cannot distinguish exercise-related information seeking from entertainment-oriented or sedentary forms of use.

#### 3.3.3 Health-related outcomes

This study examined three main health-related outcomes: self-rated health, mental wellbeing and daily functioning. All three variables were self-reported ordinal 1–5 scale measures rather than continuous clinical measures. Higher self-rated health scores indicate better perceived health. Higher mental wellbeing scores indicate fewer depressive symptoms. Higher daily functioning scores indicate fewer health-related limitations in daily life.

These outcomes were selected because physical activity and sport participation are core factors in lifestyle research. The World Health Organization has noted that regular physical activity is associated with lower risks of cardiovascular disease, diabetes, some cancers, and depressive and anxiety symptoms, as well as better overall wellbeing among adults (43). On this basis, self-rated health, mental wellbeing and daily functioning were treated as the main health-related outcomes. Cross-sectional associations were not interpreted as health effects.

Supplementary analyses also examined depressive symptom frequency and body mass index (BMI). BMI was calculated from self-reported height and weight and was used only as an exploratory health-related indicator. Because BMI relied on self-reported height and weight, and because the CGSS is not an anthropometric survey, BMI results were not used as the basis for the main conclusions.

#### 3.3.4 Additional lifestyle-overlap variables

To describe broader cross-sectional activity patterns, four additional lifestyle-overlap variables were examined: online leisure engagement, learning engagement, social engagement and friend gathering.

**Supplementary Tables S1–5** provide the supporting information for missingness, sample construction, included-vs.-excluded comparisons, measurement and coding decisions, and inverse-probability-weighted diagnostics.

These models were not interpreted as mediation analyses. They were used only to assess whether the focal association changed after adding adjacent cross-sectional correlates of everyday activity.

#### 3.3.5 Covariates

The primary model included the following covariates: age, age squared, gender, years of education, log income, self-rated health, urban-rural residence, agricultural household registration, marital status and province fixed effects.

Age was calculated as 2023 minus year of birth and restricted to 18–100 years. Years of education were derived from highest educational attainment using the coding scheme reported in Supplementary Table S4. For gender, female respondents were coded as 1 and male respondents as 0.

For heterogeneity analyses, low income was defined as personal annual income at or below the cleaned CGSS2023 income median before complete-case restriction (40,000 yuan), low education as 9 years of schooling or less, and older age as 60 years or above. These subgroup definitions are reported explicitly because they simplify more complex social positions.

### 3.4 Statistical analysis

#### 3.4.1 Primary model

Multivariable ordinary least squares (OLS) regression was first used to estimate the association between internet media use frequency and physical activity participation frequency. Lifestyle-overlap specifications were treated as descriptive covariate-adjustment models rather than mediation analyses because mediation analysis requires stronger temporal and causal assumptions (44). Heteroskedasticity-consistent standard errors were used for OLS specifications (45). The primary model was specified as follows:


$$\begin{array}{l} \begin{array}{ccc} Sport_{i} & = & \alpha +\text{β}Internet_{i}+X_{i}^{\prime }\gamma +\delta_{p}+\varepsilon_{i} \end{array} \end{array}$$ (1)

where *Sport*_*i*_ denotes physical activity participation frequency for individual *i*, *Internet*_*i*_ denotes internet media use frequency, *X*_*i*_ denotes individual-level covariates and δ_*p*_ denotes province fixed effects.

The key coefficient, β, represents the average difference in physical activity participation frequency associated with a one-level increase in internet media use frequency, conditional on the covariates.

**Supplementary Tables S6–13** report additional robustness specifications, health-related outcome models, occupation and employment checks, collinearity diagnostics, ordered-logit marginal effects for physical activity, and ordered-logit robustness models for health-related ordinal outcomes.

Because the dependent variable was an ordered 1–5 frequency measure, OLS was used as a descriptive approximation for ease of interpretation. Ordered logit was estimated as a key complementary model, and average marginal effects across outcome categories were reported to improve interpretability; full category-specific marginal effects are reported in Supplementary Table S12. Because the health-related outcomes were also ordinal five-level measures, ordered-logit robustness models for self-rated health, mental wellbeing and daily functioning are reported in Supplementary Table S13.

#### 3.4.2 Robustness analyses

Several robustness analyses were conducted, with additional robustness specifications reported in Supplementary Table S6.

First, weighted OLS was estimated using survey weights. This model assessed whether the primary findings were sensitive to weighting.

Second, an ordered logit model was estimated. Physical activity participation frequency has an ordered categorical structure, and ordered logit provides a robustness check for model form. McCullagh's (46) ordinal response model is a classical approach for analyzing ordered categorical outcomes. This model assessed whether the results depended on treating the ordered outcome as continuous in OLS.

Third, a binary logit model was estimated. This model recoded physical activity participation as regular vs. non-regular participation and assessed whether the results depended on the frequency-scale specification.

Fourth, alternative internet-related variables were used. Supplementary analyses used leisure-time internet use frequency and mobile phone ownership. Mobile phone ownership was treated as a coarse access indicator and was not considered equivalent to internet media use frequency.

Fifth, a model without self-rated health was estimated. This model assessed whether the primary result was sensitive to the inclusion of a health covariate. Self-rated health was used as a covariate only in the physical activity model. In the health-related outcome models, self-rated health was itself one of the outcomes and was therefore not included as a covariate.

#### 3.4.3 Heterogeneity analysis

Interaction models were used to examine whether the association between internet media use frequency and physical activity participation frequency differed across groups. Grouping variables included gender, urban-rural residence, income, education and age.

The interaction model was specified as follows:


$$\begin{array}{r} Sport_{i}=\alpha +\beta_{1}Internet_{i}+\beta_{2}Group_{i}+\\ \beta_{3}\left(Internet_{i}\times Group_{i}\right)+X_{i}^{\prime }\gamma +\delta_{p}+\varepsilon_{i} \end{array}$$ (2)

where β3 indicates whether the association between internet media use frequency and physical activity participation frequency was relatively stronger or weaker in a given group.

#### 3.4.4 Additional lifestyle-overlap analysis

This study further examined whether online leisure engagement, learning engagement, social engagement and friend gathering were simultaneously associated with internet media-use frequency and physical activity participation frequency.

The analysis proceeded in two steps. First, the association between internet media-use frequency and each lifestyle-overlap variable was estimated. Second, internet media-use frequency and the additional variable were included simultaneously in the physical-activity model to describe the change in the focal coefficient. Correlations and VIF diagnostics for the focal and lifestyle-overlap variables are reported in Supplementary Table S11.

This analysis was used to describe statistical overlap among variables. It was not used to establish a mechanism or causal pathway.

#### 3.4.5 Health-related outcome models

Finally, associations of internet media use frequency and physical activity participation frequency with health-related outcomes were estimated. Because these health outcomes were also ordinal 1–5 measures, the OLS models were used as descriptive approximations for comparability and ease of interpretation rather than as evidence that the response categories were strictly continuous or equally spaced. The model was specified as follows:


$$\begin{array}{l} \begin{array}{ccc} Health_{i} & = & \alpha +\text{β}Internet_{i}+\lambda Sport_{i}+Z_{i}^{\prime }\gamma +\delta_{p}+\varepsilon_{i} \end{array} \end{array}$$ (3)

where *Health*_*i*_ denotes self-rated health, mental wellbeing or daily functioning. The model included internet media use frequency and physical activity participation frequency simultaneously, and adjusted for age, age squared, gender, years of education, log income, urban-rural residence, agricultural household registration, marital status and province fixed effects.

This model was used to assess whether internet media use frequency and physical activity participation frequency co-occurred with more favorable health-related scores.

### 3.5 Sensitivity analyses for occupation, employment and working time

Because occupational position, employment status and working time may be associated with both internet use and physical activity participation, occupation- and employment-related sensitivity analyses were conducted.

Specifically, employment status, weekly working hours, current work status and International Standard Classification of Occupations (ISCO) major groups were added separately to the primary model. ISCO is the International Labour Organization's standard classification system for occupational statistics, and its major-group level is suitable for broad occupational adjustment (47).

These models were treated as sensitivity analyses rather than primary models. Some occupational variables applied only to employed respondents or to respondents with occupational codes, resulting in smaller samples than the primary analytical sample. These analyses were therefore used to assess whether the primary findings were sensitive to adjustment for occupational and employment structure (Supplementary Table S9). Employment and occupation distributions for the applicable sensitivity-analysis samples are reported in Supplementary Table S10. These analyses do not imply that occupational confounding was fully excluded.

### 3.6 Missing values, outliers and weighting

Invalid codes, values outside theoretical ranges and records that could not be converted into numeric values were set to missing. Variable-specific missingness is reported in Supplementary Table S1, sample-flow counts in Supplementary Table S2, and comparisons between included and excluded respondents in Supplementary Table S3. Income and BMI cleaning rules, educational conversion, and subgroup definitions are reported in Supplementary Table S4.

The primary model used complete-case analysis. Because included and excluded respondents differed on several observed characteristics, an inverse-probability-weighted sensitivity analysis was additionally conducted, with estimates, diagnostics and the 1st/99th percentile weight-trimming rule reported in Supplementary Table S5. Health-related outcome models used outcome-specific complete-case samples and did not include self-rated health as a covariate.

## 4 Results

### 4.1 Sample characteristics and sample selection

Table 1 presents descriptive statistics for the analytical sample. Figure 2 and Supplementary Table S2 summarize sample construction. Of 11,326 respondents in CGSS2023, 5,925 had valid information on both focal behavioral variables, and 5,621 remained after requiring complete covariate information for the primary model.

**Table 1:** Descriptive characteristics of the analytical sample.

Characteristic	N	Mean (SD) or *n* (%)	Range/category definition
Physical activity participation frequency	5,621	2.62 (1.61)	1 = never, 5 = daily
Regular physical activity	5,621	1,978 (35.2)	Daily or several times per week
Internet media-use frequency	5,621	3.62 (1.52)	1 = never, 5 = very frequently
Self-rated health	5,621	3.35 (1.13)	1 = very unhealthy, 5 = very healthy
Mental wellbeing	5,611	3.84 (1.12)	1 = always depressed/downhearted, 5 = never
Daily functioning	5,614	3.75 (1.25)	1 = always limited, 5 = never limited
Age, years	5,621	54.01 (16.47)	18–94
Female	5,621	3,055 (54.3)	Female
Age 60 years or older	5,621	2,296 (40.8)	Age >= 60
Education, years	5,621	9.30 (4.63)	0–19
Low education	5,621	3,450 (61.4)	Education years < = 9
Annual personal income, yuan	5,621	40,709.72 (74,152.17)	0–1,000,000
Log annual personal income	5,621	8.37 (3.96)	log(1 + income)
Low income	5,621	3,848 (68.5)	Income < = 40,000 yuan
Rural residence	5,621	2,567 (45.7)	Rural residence
Agricultural hukou	5,621	3,863 (68.7)	Agricultural hukou or resident hukou formerly agricultural
Married/cohabiting	5,621	4,012 (71.4)	Married or cohabiting

Values are mean (SD) for continuous and ordinal variables and n (%) for binary variables. The analytical sample includes respondents with complete data for the primary physical-activity model (*N* = 5,621). Low income was defined as income at or below the cleaned CGSS2023 income median before complete-case restriction (40,000 yuan).

Supplementary Table S1 shows that missingness was concentrated in the focal behavioral variables and some lifestyle-overlap variables: physical activity participation frequency was missing for 38.2% of respondents, internet media-use frequency for 38.4%, learning engagement for 50.8%, social engagement for 50.7%, and friend gathering for 38.3%.

Included and excluded respondents differed on several observed characteristics, including age, education, income, rural residence and internet use, indicating that sample selection should be considered when interpreting the estimates.

### 4.2 Descriptive association between internet media use frequency and physical activity participation frequency

**Figure 3:**
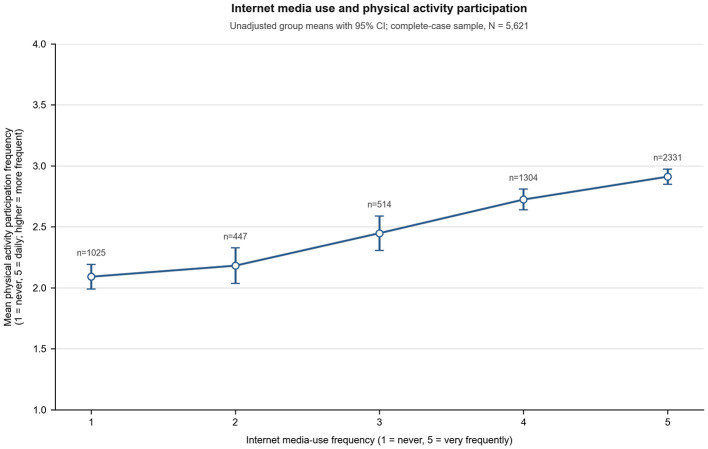
Association between internet media-use frequency and physical activity participation frequency.

Figure 3 shows the unadjusted relationship between levels of internet media-use frequency and mean physical activity participation frequency. Mean physical activity participation increased as internet media-use frequency rose from level 1 to level 5.

Specifically, respondents with internet media use frequency at level 1 had a mean physical activity participation frequency of 2.09. The corresponding means were 2.17 at level 2, 2.45 at level 3, 2.72 at level 4 and 2.92 at level 5.

Figure 3 reports unadjusted group means. It therefore indicates descriptive differences across internet media-use frequency groups before adjustment for covariates. A by-residence descriptive version is shown in Supplementary Figure S2.

### 4.3 Main model and robustness analyses

Table 2 reports multivariable model results for the association between internet media use frequency and physical activity participation frequency.

**Table 2:** Main and robustness models.

Model	Dependent variable	Key coefficient	Estimate (SE; 95% CI)	*p*-value	*N*	R^2^/log likelihood/statistic	Controls	Province FE	Model details
OLS	Physical activity participation frequency	Internet media-use frequency	0.084^***^ (0.018; 0.049 to 0.119)	< 0.001	5,621	0.18	Yes	Yes	Main model
Weighted OLS	Physical activity participation frequency	Internet media-use frequency	0.079^***^ (0.019; 0.042 to 0.116)	< 0.001	5,621	0.187	Yes	Yes	Survey weight applied
Ordered logit	Physical activity participation frequency	Internet media-use frequency	0.144^***^ (0.022; 0.101 to 0.187)	< 0.001	5,621	−7,791.673	Yes	No	Ordered outcome robustness; province FE omitted for computational stability
Binary logit	Regular physical activity	Internet media-use frequency	0.116^***^ (0.026; 0.065 to 0.167)	< 0.001	5,621	—	Yes	Yes	AME for +1 level = 0.013

^***^Denotes *p* < 0.001.

In the primary OLS model, internet media-use frequency was positively but modestly associated with physical activity participation frequency. After adjustment for covariates and province fixed effects, a one-level increase in internet media-use frequency was associated with a 0.084-point higher physical activity participation score.

The direction of the association was consistent in weighted OLS, ordered logit and binary logit specifications. Ordered-logit marginal effects indicated that a one-level increase in internet media use was associated with a 1.8-percentage-point lower probability of being in the lowest physical-activity category and a 1.1-percentage-point higher probability of being in the highest category (Supplementary Table S12).

An inverse-probability-weighted sensitivity analysis produced a similar estimate (b = 0.094, SE = 0.018, 95% CI 0.058 to 0.130), suggesting robustness to reweighting for observed predictors of analytical-sample inclusion while not excluding bias from unobserved missingness mechanisms (Supplementary Table S5).

The binary logit model redefined the outcome as whether respondents reported regular physical activity. The coefficient for internet media use frequency was 0.116 (s.e. = 0.026, *p* < 0.001). The average marginal difference was 0.013. Thus, a one-level higher internet media use frequency was associated with a 1.3 percentage-point higher predicted probability of regular physical activity. This result indicates that the direction of the main association remained consistent when physical activity participation was recoded as a binary outcome.

Overall, Table 2 shows that the positive association between internet media use frequency and physical activity participation frequency was consistent across OLS, weighted OLS, ordered logit and binary logit models.

### 4.4 Demographic and socioeconomic heterogeneity

Table 3 reports the heterogeneity analyses. These models tested whether the association between internet media use frequency and physical activity participation frequency differed by gender, urban-rural residence, income, education and age. A visual summary of the interaction coefficients and 95% confidence intervals is provided in Supplementary Figure S3.

**Table 3:** Subgroup heterogeneity.

Subgroup dimension	Interaction term	Reference-group internet coefficient (SE; 95% CI)	Interaction coefficient (SE; 95% CI)	Interaction *p*-value	*N*
Female	Internet media-use frequency × female	0.095^***^ (0.023; 0.050 to 0.140)	−0.019 (0.027; −0.072 to 0.034)	0.47	5,621
Rural residence	Internet media-use frequency × rural residence	0.089^***^ (0.025; 0.040 to 0.138)	−0.008 (0.029; −0.065 to 0.049)	0.785	5,621
Low income	Internet media-use frequency × low income	0.009 (0.036; −0.062 to 0.080)	0.093^*^ (0.038; 0.019 to 0.167)	0.013	5,621
Low education	Internet media-use frequency × low education	0.120^***^ (0.035; 0.051 to 0.189)	−0.045 (0.038; −0.119 to 0.029)	0.231	5,621
Age 60+	Internet media-use frequency × age 60+	0.053^*^ (0.025; 0.004 to 0.102)	0.054 (0.032; −0.009 to 0.117)	0.086	5,621

^*^Denotes *p* < 0.05 and ^***^denotes *p* < 0.001.

For gender, the interaction term between internet media use frequency and being female was −0.019 (s.e. = 0.027, *p* = 0.470). This result showed no clear evidence of gender heterogeneity. Under this model specification, the association between internet media use frequency and physical activity participation frequency did not differ statistically between men and women.

For urban-rural residence, the interaction term between internet media use frequency and rural residence was −0.008 (s.e. = 0.029, *p* = 0.785). This result showed no clear evidence of urban-rural heterogeneity.

For income, the low-income interaction term was positive and statistically significant. The interaction between internet media use frequency and low-income status was 0.093 (s.e. = 0.038, *p* = 0.013). This result indicates that the positive association between internet media use frequency and physical activity participation frequency was stronger among low-income respondents. The coefficient for internet media use was 0.009 in the non-low-income group, whereas the corresponding slope was approximately 0.102 in the low-income group.

For age, the interaction term for respondents aged 60 years or older was positive but did not reach conventional statistical significance (interaction coefficient = 0.054, s.e. = 0.032, *p* = 0.086). This result was treated as exploratory rather than evidence of a clear age-specific difference.

For education, the interaction term between internet media use frequency and low education was −0.045 (s.e. = 0.038, *p* = 0.231). This result showed no clear evidence of educational heterogeneity.

### 4.5 Additional lifestyle-overlap analysis

Table 4 reports exploratory lifestyle-overlap models. Descriptive attenuation shares are summarized in Supplementary Figure S4.

**Table 4:** Exploratory lifestyle-overlap models.

Additional lifestyle-overlap variable	Association with internet media-use frequency (SE; 95% CI)	Association with physical activity participation frequency (SE; 95% CI)	Coefficient for internet media-use frequency after adding lifestyle-overlap variable (SE; 95% CI)	Descriptive attenuation share	*N*
Online leisure engagement	0.819^***^ (0.012; 0.795 to 0.843)	0.053^*^ (0.023; 0.008 to 0.098)	0.041 (0.026; −0.010 to 0.092)	0.516	5,379
Learning engagement	0.095^***^ (0.012; 0.071 to 0.119)	0.311^***^ (0.021; 0.270 to 0.352)	0.055^**^ (0.018; 0.020 to 0.090)	0.352	5,306
Social engagement	0.078^***^ (0.013; 0.053 to 0.103)	0.112^***^ (0.019; 0.075 to 0.149)	0.075^***^ (0.018; 0.040 to 0.110)	0.105	5,311
Friend gathering	0.065^***^ (0.011; 0.043 to 0.087)	0.211^***^ (0.023; 0.166 to 0.256)	0.070^***^ (0.018; 0.035 to 0.105)	0.171	5,382

^*^Denotes *p* < 0.05, ^**^Denotes *p* < 0.01, and ^***^denotes *p* < 0.001.

Online leisure engagement, learning engagement, social engagement and friend gathering were each positively associated with both internet media-use frequency and physical activity participation frequency.

After adding these variables, the coefficient for internet media-use frequency was smaller than in the baseline model, with the largest descriptive attenuation observed after adding online leisure engagement and learning engagement.

Online leisure engagement showed the strongest overlap with internet media-use frequency. Its association with internet media-use frequency was 0.819 (s.e. = 0.012, *p* < 0.001); after online leisure engagement was added to the physical-activity model, the internet coefficient declined to 0.041 (s.e. = 0.026), corresponding to a descriptive attenuation share of 51.6%.

These models are interpreted as cross-sectional overlap analyses rather than mediation tests.

Learning engagement was positively associated with internet media-use frequency, with a coefficient of 0.095 (s.e. = 0.012, *p* < 0.001). After learning engagement was added to the physical-activity model, its coefficient was 0.311 (s.e. = 0.021, *p* < 0.001). The coefficient for internet media-use frequency was 0.055 (s.e. = 0.018, *p* < 0.01), a descriptive attenuation of 35.2%.

Social engagement was positively associated with internet media-use frequency, with a coefficient of 0.078 (s.e. = 0.013, *p* < 0.001). After social engagement was added to the physical-activity model, its coefficient was 0.112 (s.e. = 0.019, *p* < 0.001). The coefficient for internet media-use frequency was 0.075 (s.e. = 0.018, *p* < 0.001), a descriptive attenuation of 10.5%.

Friend gathering was positively associated with internet media-use frequency, with a coefficient of 0.065 (s.e. = 0.011, *p* < 0.001). After friend gathering was added to the physical-activity model, its coefficient was 0.211 (s.e. = 0.023, *p* < 0.001). The coefficient for internet media-use frequency was 0.070 (s.e. = 0.018, *p* < 0.001), a descriptive attenuation of 17.1%.

### 4.6 Internet media use frequency, physical activity participation frequency and health-related outcomes

**Figure 4:**
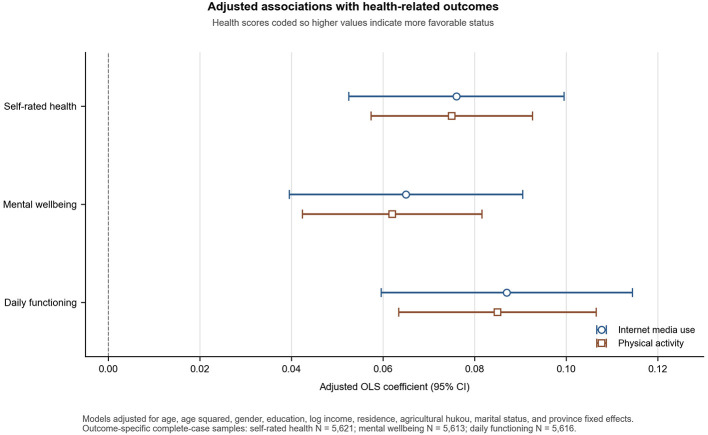
Associations of internet media use and physical activity participation with health-related outcomes.

Table 5 reports model results for the associations of internet media use frequency and physical activity participation frequency with health-related outcomes. Figure 4 presents adjusted coefficients and 95% confidence intervals for the main health-outcome OLS models. Weighted health-outcome models, additional health-related outcome models and ordered-logit health-outcome robustness models are reported in Supplementary Tables S7, S8 and S13.

**Table 5:** Health-related outcome models.

Health-related outcome	Coefficient for internet media-use frequency (SE; 95% CI)	Coefficient for physical activity participation frequency (SE; 95% CI)	*N*	R^2^
Self-rated health	0.076^***^ (0.012; 0.052 to 0.100)	0.075^***^ (0.009; 0.057 to 0.093)	5,621	0.204
Mental wellbeing	0.065^***^ (0.013; 0.040 to 0.090)	0.062^***^ (0.010; 0.042 to 0.082)	5,613	0.092
Daily functioning	0.087^***^ (0.014; 0.060 to 0.114)	0.085^***^ (0.011; 0.063 to 0.107)	5,616	0.185

The models in Table 5 use outcome-specific complete-case samples. Mental wellbeing and daily functioning models did not require valid self-rated health because self-rated health was not included as a covariate in these health-related outcome models. ^***^Denotes *p* < 0.001.

In the self-rated health model, the coefficient for internet media use frequency was 0.076 (s.e. = 0.012, *p* < 0.001). The coefficient for physical activity participation frequency was 0.075 (s.e. = 0.009, *p* < 0.001). The model included 5,621 respondents and had an R^2^ of 0.204. Conditional on covariates, respondents with higher internet media use frequency and higher physical activity participation frequency reported higher self-rated health scores.

In the daily functioning model, the coefficient for internet media use frequency was 0.087 (s.e. = 0.014, *p* < 0.001). The coefficient for physical activity participation frequency was 0.085 (s.e. = 0.011, *p* < 0.001). The model included 5,616 respondents and had an R^2^ of 0.185. These results show that both internet media use frequency and physical activity participation frequency were associated with higher daily functioning scores.

Figure 4 shows that the coefficients for internet media-use frequency and physical activity participation frequency were positive across all three health-related outcomes. The coefficients for the two variables were similar in magnitude across the three models. In the self-rated health model, the coefficients were 0.076 and 0.075, respectively. In the mental wellbeing model, the corresponding coefficients were 0.065 and 0.062, indicating small positive associations with less frequent depressive mood. In the daily functioning model, they were 0.087 and 0.085. Ordered-logit robustness models for these three ordinal outcomes showed the same direction of association (Supplementary Table S13).

These results indicate that, in the complete-case sample, internet media use frequency and physical activity participation frequency both co-occurred with more favorable self-reported health-related scores.

## 5 Discussion and conclusion

Using CGSS2023, this study examined the cross-sectional association between internet media-use frequency and physical activity participation frequency among Chinese adults, together with social-group heterogeneity and secondary health-related associations. Across model specifications, respondents with more frequent internet media use tended to report slightly more frequent physical activity participation.

Previous research has shown that physical activity participation is shaped by individual characteristics, social relationships, socioeconomic resources and environmental conditions (48–51). Ecological models similarly emphasize that health behaviors are not determined solely by individual intention, but are embedded in social, environmental and institutional contexts (52). As digital life continues to expand, more empirical evidence is needed on whether internet use co-occurs with physical activity participation, particularly among Chinese adults. Chinese evidence on digital information acquisition, digital lifestyle, social participation, rural and low-income groups, digital skills and health behaviors also suggests that the social meaning of internet use varies across population groups and activity domains (53–60). Based on the complete-case sample from CGSS2023, this study examined cross-sectional associations among internet media use frequency, physical activity participation frequency and health-related outcomes. Internet media use frequency showed a stable positive association with physical activity participation frequency. The direction of this association was consistent across OLS, weighted OLS, ordered logit and binary logit models. This finding accords with previous studies reporting positive associations between internet use and physical activity participation among middle-aged and older adults in China (13–15).

The positive association should be interpreted as a modest descriptive pattern rather than as evidence that internet use promotes exercise. Because the CGSS measure captures overall frequency rather than use content, frequent internet media use may reflect information access, social connection or broader activity engagement, but the data cannot identify which online activities are relevant.

The clearest subgroup difference concerned income. The association was stronger among low-income respondents, which may suggest that internet use is more valuable where offline sport resources, paid instruction and facility access are more constrained. Online platforms may reduce information barriers, provide low-cost exercise content and support social or community-based encouragement for activity. These explanations remain hypotheses rather than demonstrated mechanisms. No clear heterogeneity was observed by urban-rural residence. In the Chinese context, this null finding should be read alongside policies and infrastructure changes that have narrowed some dimensions of the urban-rural digital divide, including rural broadband expansion and the Digital Village Strategy, while unequal access to community sport services and fitness facilities may still vary within both urban and rural areas. The broad residence indicator used here may therefore be too coarse to capture these policy-relevant differences.

The lifestyle-overlap analysis further showed that online leisure engagement, learning engagement, social engagement and friend gathering overlapped statistically with both internet media-use frequency and physical activity participation frequency. Correlation and VIF diagnostics showed conceptual proximity between internet media-use frequency and online leisure engagement, but did not indicate severe multicollinearity among the focal lifestyle-overlap covariates (Supplementary Table S11). Coefficient attenuation after these variables were added is interpreted descriptively and should not be read as evidence of causal mechanisms or mediation.

The health-related outcomes broaden the descriptive public-health relevance of the study. Internet media-use frequency and physical activity participation frequency were each associated with more favorable self-rated health, mental wellbeing and daily functioning, but these associations were small and cross-sectional.

This study has several limitations. First, its cross-sectional design precludes assessment of temporal ordering among internet use, physical activity and health-related outcomes. Second, the measures of internet use and physical activity capture frequency only, rather than duration, intensity, content or context. Third, several focal behavioral variables had substantial missingness. Although the IPW sensitivity analysis yielded results consistent with the primary models, it adjusts only for observed predictors of analytical-sample inclusion. It therefore cannot rule out bias arising from unobserved missingness mechanisms. Multiple imputation was not used because the variables with the largest missingness were ordinal focal behavioral measures. Imputing these variables would have required stronger distributional and substantive assumptions than the reweighting approach adopted here. Finally, some subgroup indicators necessarily simplify more complex socioeconomic positions. The income and BMI exclusions were validity screens rather than winsorization procedures: income values up to 1,000,000 yuan were retained as the extreme upper tail of the cleaned distribution, and BMI was restricted only by a broad physiological plausibility range.

## Data Availability

Publicly available datasets were analyzed in this study. The data were obtained from the 2023 Chinese General Social Survey (CGSS2023), conducted by the National Survey Research Center at Renmin University of China. The CGSS2023 dataset is available through the Chinese National Survey Data Archive (CNSDA): https://www.cnsda.org/index.php?id=11256439&r=projects/view. Access to the dataset may require registration and compliance with the data-use requirements of the data provider.
