# Supplementary material for: Internet media use, physical activity participation, and health-related outcomes among Chinese adults: evidence from CGSS2023
Source: Front Public Health. 2026 Jun 15;14:1873007. doi: 10.3389/fpubh.2026.1873007 (PMC13310743; doi:10.3389/fpubh.2026.1873007)

Supplementary Material

**Internet Media Use, Physical Activity Participation, and Health-Related Outcomes Among Chinese Adults: Evidence from CGSS2023**

Supplementary Tables

**Supplementary Table S1. Variable missingness**

| **Variable** | **Analysis variable** | **Non-missing N** | **Missing N** | **Missing (%)** |
| --- | --- | --- | --- | --- |
| Physical activity participation frequency | sport_freq | 7003 | 4323 | 38.2 |
| Internet media-use frequency | internet_media_freq | 6976 | 4350 | 38.4 |
| Age | age | 11242 | 84 | 0.7 |
| Female | female | 11326 | 0 | 0.0 |
| Education years | edu_years | 11320 | 6 | 0.1 |
| Log income | ln_income | 10608 | 718 | 6.3 |
| Self-rated health | self_rated_health | 11322 | 4 | 0.0 |
| Rural residence | rural_residence | 11256 | 70 | 0.6 |
| Agricultural hukou | agricultural_hukou | 11261 | 65 | 0.6 |
| Married/cohabiting | married | 11322 | 4 | 0.0 |
| Province | province | 11318 | 8 | 0.1 |
| Learning engagement | learning_engagement | 5576 | 5750 | 50.8 |
| Social engagement | social_engagement | 5582 | 5744 | 50.7 |
| Friend gathering | friend_gathering | 6993 | 4333 | 38.3 |
| Mental wellbeing | mental_wellbeing | 11288 | 38 | 0.3 |
| Daily functioning | daily_functioning | 11296 | 30 | 0.3 |

**Note:** Missing rates are calculated using the original CGSS2023 sample of 11,326 respondents. Percentages are shown to one decimal place.

**Supplementary Table S2. Sample flow**

| **Step** | **N** | **Excluded since previous step** | **Percent of original sample retained** |
| --- | --- | --- | --- |
| Original CGSS2023 sample | 11326 |  | 100.0 |
| Valid physical activity and internet media-use measures | 5925 | 5401 | 52.3 |
| Complete covariate data among respondents with valid main variables | 5621 | 304 | 49.6 |

**Note:** The decomposition separates focal-variable availability from subsequent complete-case restrictions. Percentages are shown to one decimal place.

**Supplementary Table S3. Included versus excluded respondents**

| **Variable** | **Included N** | **Included mean/proportion** | **Included SD** | **Excluded N** | **Excluded mean/proportion** | **Excluded SD** | **Difference** | **SMD** | **Welch p value** |
| --- | --- | --- | --- | --- | --- | --- | --- | --- | --- |
| Physical activity frequency | 5621 | 2.617 | 1.612 | 1382 | 3.149 | 1.343 | -0.532 | -0.358 | <0.001 |
| Regular physical activity | 5621 | 0.352 | 0.478 | 1382 | 0.458 | 0.498 | -0.106 | -0.217 | <0.001 |
| Internet media-use frequency | 5621 | 3.617 | 1.523 | 1355 | 4.258 | 1.122 | -0.641 | -0.479 | <0.001 |
| Age | 5621 | 54.010 | 16.468 | 5621 | 41.494 | 14.476 | 12.516 | 0.807 | <0.001 |
| Female | 5621 | 0.543 | 0.498 | 5705 | 0.360 | 0.480 | 0.183 | 0.374 | <0.001 |
| Education years | 5621 | 9.295 | 4.627 | 5699 | 13.666 | 3.588 | -4.370 | -1.056 | <0.001 |
| Log income | 5621 | 8.369 | 3.961 | 4987 | 9.815 | 3.689 | -1.446 | -0.378 | <0.001 |
| Self-rated health | 5621 | 3.354 | 1.126 | 5701 | 3.421 | 0.953 | -0.066 | -0.064 | <0.001 |
| Mental wellbeing | 5611 | 3.840 | 1.115 | 5677 | 3.461 | 0.990 | 0.379 | 0.359 | <0.001 |
| Daily functioning | 5614 | 3.747 | 1.247 | 5682 | 3.603 | 1.003 | 0.144 | 0.127 | <0.001 |
| Rural residence | 5621 | 0.457 | 0.498 | 5635 | 0.168 | 0.374 | 0.289 | 0.656 | <0.001 |
| Agricultural hukou | 5621 | 0.687 | 0.464 | 5640 | 0.549 | 0.498 | 0.138 | 0.287 | <0.001 |
| Married/cohabiting | 5621 | 0.714 | 0.452 | 5701 | 0.649 | 0.477 | 0.064 | 0.139 | <0.001 |
| Currently working | 5312 | 0.473 | 0.499 | 271 | 0.391 | 0.489 | 0.082 | 0.166 | 0.007 |
| Worked last week | 5312 | 0.455 | 0.498 | 271 | 0.373 | 0.484 | 0.082 | 0.167 | 0.007 |
| Weekly work hours | 2468 | 48.503 | 20.948 | 96 | 47.896 | 23.690 | 0.607 | 0.027 | 0.805 |

**Note:** Excluded respondents are those not retained in the complete-case analytical sample. Means, SDs, differences and SMDs are reported to three decimal places; p values are reported as <0.001 or to three decimal places.

**Supplementary Table S4. Measurement and coding**

| **Construct** | **CGSS2023 item(s)** | **Item wording, translated from CGSS2023 codebook** | **Role in analysis** | **Operationalization** | **Rationale / limitation** |
| --- | --- | --- | --- | --- | --- |
| Physical activity participation frequency | A309 / a3009 | In the past year, how often did you engage in physical exercise during leisure time? | Main behavioral outcome | Original A30 scale: 1 = every day, 2 = several times a week, 3 = several times a month, 4 = several times a year or less, 5 = never. Reverse-coded so that 1 = never and 5 = daily. | Frequency-only measure; does not capture duration, intensity, activity type, occupational activity, or WHO guideline adherence. |
| Regular physical activity | A309 / a3009 | In the past year, how often did you engage in physical exercise during leisure time? | Robustness outcome | Binary recode: original 1 = every day or 2 = several times a week coded as 1; original 3-5 coded as 0. | Cutoff separates at-least-weekly participation (daily or several times per week) from less frequent participation; this threshold provides a transparent binary robustness check rather than a WHO guideline classification because duration and intensity are unavailable. |
| Internet media-use frequency | A285 / a285 | In the past year, how often did you use the internet as a medium, including going online through mobile phones, computers, tablets, smart wearable devices, or similar devices? | Main exposure | A28 scale: 1 = never, 2 = rarely, 3 = sometimes, 4 = often, 5 = very frequently. Higher values indicate more frequent internet media use. | Does not distinguish fitness information, online courses, news, social media, entertainment, gaming, or passive browsing. |
| Leisure-time internet use | A3012 / a3012 | In the past year, how often did you go online during leisure time? | Additional lifestyle-overlap covariate | Original A30 scale reverse-coded so higher values indicate more frequent leisure-time internet use. | Conceptually related to internet media use; used only as a descriptive overlap covariate, not a mediator. |
| Learning engagement | A313 / a313 | In the past year, how often did you engage in learning or self-improvement during leisure time? | Additional lifestyle-overlap covariate | A31 scale: 1 = never, 2 = rarely, 3 = sometimes, 4 = often, 5 = very frequently. Higher values indicate more frequent learning engagement. | Used as an additional covariate, not a causal mediator. |
| Social engagement | A311 / a311 | In the past year, how often did you engage in socializing or visiting during leisure time? | Additional lifestyle-overlap covariate | A31 scale: 1 = never, 2 = rarely, 3 = sometimes, 4 = often, 5 = very frequently. Higher values indicate more frequent social engagement. | Used as an additional covariate, not a causal mediator. |
| Friend gathering | A307 / a3007 | In the past year, how often did you gather with friends during leisure time? | Additional lifestyle-overlap covariate | Original A30 scale reverse-coded so higher values indicate more frequent friend gathering. | Captures offline social participation frequency. |
| Self-rated health | A15 / a15 | How would you rate your current physical health? | Health-related outcome and covariate | 1 = very unhealthy, 2 = relatively unhealthy, 3 = average, 4 = relatively healthy, 5 = very healthy. | Ordinal health measure. Included as a covariate in physical-activity models and examined descriptively as a health-related outcome. |
| Daily functioning | A16 / a16 | During the past four weeks, how often did health problems affect your work or other daily activities? | Secondary health-related outcome | 1 = always, 2 = often, 3 = sometimes, 4 = rarely, 5 = never. Higher values indicate fewer health-related activity limitations. | Ordinal outcome interpreted descriptively. |
| Mental wellbeing | A17 / a17 | During the past four weeks, how often did you feel depressed or downhearted? | Secondary health-related outcome | 1 = always, 2 = often, 3 = sometimes, 4 = rarely, 5 = never. Higher values indicate less frequent depressive mood. | Ordinal outcome interpreted descriptively. |
| Education years | A7a / a7a | What is your highest level of educational attainment? | Socioeconomic covariate and subgroup base variable | Mapped as: no education = 0, literacy class/private school = 2, primary school = 6, junior middle school = 9, vocational high school/general high school/technical secondary school/technical school = 12, adult or regular college = 15, adult or regular undergraduate degree = 16, postgraduate or above = 19 years; other/refusal values set to missing. | Approximation of years of schooling from detailed CGSS education categories. |
| Low education | A7a / a7a | What is your highest level of educational attainment? | Subgroup variable | Education years <= 9 coded as low education; >9 coded as higher education. | Cutoff corresponds approximately to completion of compulsory schooling in China. |
| Income and log income | A8a / a8a | What was your total personal income in 2022? | Socioeconomic covariate and subgroup base variable | Annual personal income retained from 0 to 1,000,000 yuan; special missing/nonresponse codes excluded; log income computed as log(1 + income). | Upper validity rule removes implausibly high or miscoded values while retaining zero income. The 1,000,000-yuan ceiling corresponds to the extreme upper tail of the cleaned distribution (99th percentile = 500,000; 99.5th percentile = 600,000; 99.9th percentile = 1,000,000) and was used as a validity screen, not winsorization. |
| Low income | A8a / a8a | What was your total personal income in 2022? | Subgroup variable | Respondents with income at or below the cleaned CGSS2023 income median before complete-case restriction (40,000 yuan) were coded as low income; those above this threshold were coded as higher income. | This threshold preserves a transparent, pre-specified within-dataset heterogeneity contrast; after complete-case restriction, the final analytical sample is not split 50/50. |
| BMI | A13/A14 / a13/a14 | Current height in centimeters and current weight in jin. | Supplementary health-related outcome | BMI computed from self-reported height and weight after converting weight from jin to kilograms; values outside 10-60 set to missing. | Restriction removes implausible anthropometric values using a broad physiological plausibility screen. In the cleaned data, the observed maximum was 45.0 and the 99.9th percentile was 37.8, so the 10-60 rule functions as a conservative validity check rather than winsorization. |

**Note:** Item wording was translated from the CGSS2023 codebook. Negative special codes were treated as missing where applicable. Income and BMI cleaning rules were validity screens rather than winsorization procedures.

**Supplementary Table S5. IPW sensitivity and diagnostics**

| **Model** | **Estimate** | **SE** | **95% CI** | **p value** | **N** |
| --- | --- | --- | --- | --- | --- |
| Primary OLS complete-case model | 0.084 | 0.018 | [0.049, 0.119] | <0.001 | 5621 |
| IPW sensitivity model | 0.094 | 0.018 | [0.058, 0.130] | <0.001 | 5621 |
| IPW diagnostics | Mean predicted inclusion probability | 0.496 |  |  |  |
| IPW diagnostics | Observed inclusion proportion | 0.496 |  |  |  |
| IPW diagnostics | Minimum predicted inclusion probability | 1.106×10-54 |  |  |  |
| IPW diagnostics | Maximum predicted inclusion probability | 0.997 |  |  |  |
| IPW diagnostics | 1st percentile IPW before trimming | 1.011 |  |  |  |
| IPW diagnostics | 99th percentile IPW before trimming | 8.074 |  |  |  |

**Note:** Predicted inclusion probabilities were estimated in the full CGSS2023 sample. Analytical IPW weights were trimmed at the 1st and 99th percentiles before fitting the weighted model; the extremely small minimum predicted inclusion probability is reported in scientific notation as a selection-model diagnostic, not as an untrimmed estimation weight.

**Supplementary Table S6. Additional robustness checks**

| **Model** | **Dependent variable** | **Key coefficient** | **Estimate**  **(SE)** | **p value** | **N** | **R2 / statistic** | **Controls** | **Province FE** | **Notes** |
| --- | --- | --- | --- | --- | --- | --- | --- | --- | --- |
| Alternative internet measure | Physical activity frequency | internet_leisure_freq | 0.082*** (0.015) | <0.001 | 5603 | 0.180 | Yes | Yes | Leisure-time internet use |
| Mobile phone ownership | Physical activity frequency | own_mobile_phone | 0.162 (0.110) | 0.142 | 5643 | 0.175 | Yes | Yes | Device access indicator |
| No self-rated-health control | Physical activity frequency | internet_media_freq | 0.097*** (0.018) | <0.001 | 5623 | 0.170 | Yes | Yes | Health control excluded |

**Note:** Robustness checks examine alternative operationalizations and model specifications. Estimates, SEs and R2/statistics are rounded to three decimal places; p values are reported as <0.001 or to three decimal places.

**Supplementary Table S7. Weighted health models**

| **Health-related outcome** | **Internet use coefficient** | **Physical activity coefficient** | **N** | **R2** |
| --- | --- | --- | --- | --- |
| Self-rated health | 0.076*** (0.014) | 0.080*** (0.010) | 5621 | 0.230 |
| Mental wellbeing | 0.059*** (0.015) | 0.067*** (0.011) | 5613 | 0.091 |
| Daily functioning | 0.086*** (0.016) | 0.085*** (0.012) | 5616 | 0.195 |

**Note:** Weighted models use CGSS survey weights where available. Coefficients and R2 values are rounded to three decimal places.

**Supplementary Table S8. Additional health models**

| **Outcome** | **Internet use coefficient** | **Physical activity coefficient** | **N** | **R2** |
| --- | --- | --- | --- | --- |
| Depressive mood frequency | -0.065*** (0.013) | -0.062*** (0.010) | 5613 | 0.092 |
| BMI | 0.187*** (0.040) | 0.054+ (0.032) | 5581 | 0.082 |

**Note:** Additional health outcomes are interpreted as secondary cross-sectional associations. Coefficients and R2 values are rounded to three decimal places.

**Supplementary Table S9. Employment robustness checks**

| **Model** | **Dependent variable** | **Key coefficient** | **Estimate**  **(SE)** | **p value** | **N** | **R2 / statistic** | **Controls** | **Province FE** | **Notes** |
| --- | --- | --- | --- | --- | --- | --- | --- | --- | --- |
| Main model + employment status | Physical activity frequency | internet_media_freq | 0.079*** (0.018) | <0.001 | 5312 | 0.180 | Yes | Yes | Adds work-experience/current employment status; full applicable sample |
| Main model + weekly work hours | Physical activity frequency | internet_media_freq | 0.086** (0.028) | 0.002 | 2468 | 0.182 | Yes | Yes | Controls weekly work hours; valid-hours subsample |
| Main model + current work status | Physical activity frequency | internet_media_freq | 0.064 (0.042) | 0.128 | 1706 | 0.133 | Yes | Yes | Controls current work status; applicable employed/nonfarm subsample |
| Main model + ISCO major group | Physical activity frequency | internet_media_freq | 0.087+ (0.044) | 0.051 | 1417 | 0.149 | Yes | Yes | Controls ISCO major group; nonfarm occupation-coded subsample |

**Note:** Employment and occupation adjustments evaluate whether the main estimate is sensitive to labor-market covariates. Estimates, SEs and R2/statistics are rounded to three decimal places; p values are reported as <0.001 or to three decimal places.

**Supplementary Table S10. Employment and occupation distributions**

| **Variable** | **Sample** | **Category** | **N** | **Percent within sample** |
| --- | --- | --- | --- | --- |
| Employment status / work experience | Excluded from main analytical sample | 1 | 66 | 24.4 |
| Employment status / work experience | Excluded from main analytical sample | 2 | 14 | 5.2 |
| Employment status / work experience | Excluded from main analytical sample | 3 | 36 | 13.3 |
| Employment status / work experience | Excluded from main analytical sample | 4 | 46 | 17.0 |
| Employment status / work experience | Excluded from main analytical sample | 5 | 79 | 29.2 |
| Employment status / work experience | Excluded from main analytical sample | 6 | 30 | 11.1 |
| Employment status / work experience | Included in main analytical sample | 1 | 1709 | 32.2 |
| Employment status / work experience | Included in main analytical sample | 2 | 333 | 6.3 |
| Employment status / work experience | Included in main analytical sample | 3 | 703 | 13.2 |
| Employment status / work experience | Included in main analytical sample | 4 | 739 | 13.9 |
| Employment status / work experience | Included in main analytical sample | 5 | 1508 | 28.4 |
| Employment status / work experience | Included in main analytical sample | 6 | 320 | 6.0 |
| Current occupation category | Excluded from main analytical sample | 1 | 343 | 6.3 |
| Current occupation category | Excluded from main analytical sample | 2 | 204 | 3.8 |
| Current occupation category | Excluded from main analytical sample | 3 | 344 | 6.4 |
| Current occupation category | Excluded from main analytical sample | 4 | 412 | 7.6 |
| Current occupation category | Excluded from main analytical sample | 5 | 754 | 13.9 |
| Current occupation category | Excluded from main analytical sample | 6 | 260 | 4.8 |
| Current occupation category | Excluded from main analytical sample | 7 | 50 | 0.9 |
| Current occupation category | Excluded from main analytical sample | 8 | 529 | 9.8 |
| Current occupation category | Excluded from main analytical sample | 9 | 339 | 6.3 |
| Current occupation category | Excluded from main analytical sample | 10 | 240 | 4.4 |
| Current occupation category | Excluded from main analytical sample | 11 | 67 | 1.2 |
| Current occupation category | Excluded from main analytical sample | 12 | 288 | 5.3 |
| Current occupation category | Excluded from main analytical sample | 13 | 28 | 0.5 |
| Current occupation category | Excluded from main analytical sample | 14 | 338 | 6.2 |
| Current occupation category | Excluded from main analytical sample | 15 | 538 | 9.9 |
| Current occupation category | Excluded from main analytical sample | 16 | 487 | 9.0 |
| Current occupation category | Excluded from main analytical sample | 17 | 188 | 3.5 |
| Current occupation category | Included in main analytical sample | 1 | 16 | 5.2 |
| Current occupation category | Included in main analytical sample | 2 | 14 | 4.5 |
| Current occupation category | Included in main analytical sample | 3 | 17 | 5.5 |
| Current occupation category | Included in main analytical sample | 4 | 31 | 10.0 |
| Current occupation category | Included in main analytical sample | 5 | 52 | 16.8 |
| Current occupation category | Included in main analytical sample | 6 | 11 | 3.6 |
| Current occupation category | Included in main analytical sample | 7 | 3 | 1.0 |
| Current occupation category | Included in main analytical sample | 8 | 33 | 10.7 |
| Current occupation category | Included in main analytical sample | 9 | 15 | 4.9 |
| Current occupation category | Included in main analytical sample | 10 | 14 | 4.5 |
| Current occupation category | Included in main analytical sample | 11 | 1 | 0.3 |
| Current occupation category | Included in main analytical sample | 12 | 13 | 4.2 |
| Current occupation category | Included in main analytical sample | 13 | 4 | 1.3 |
| Current occupation category | Included in main analytical sample | 14 | 16 | 5.2 |
| Current occupation category | Included in main analytical sample | 15 | 37 | 12.0 |
| Current occupation category | Included in main analytical sample | 16 | 17 | 5.5 |
| Current occupation category | Included in main analytical sample | 17 | 15 | 4.9 |

**Note:** Distributions are provided to document the additional labor-market variables used in robustness checks. Percentages are shown to one decimal place.

**Supplementary Table S11. Correlation and VIF diagnostics**

Panel A. Spearman correlation matrix

| **Variable** | **Internet media use frequency** | **Physical activity participation frequency** | **Online leisure engagement** | **Learning engagement** | **Social engagement** | **Friend gathering** |
| --- | --- | --- | --- | --- | --- | --- |
| Internet media use frequency | 1.000 | 0.217 | 0.762 | 0.368 | 0.143 | 0.299 |
| Physical activity participation frequency | 0.217 | 1.000 | 0.223 | 0.370 | 0.098 | 0.256 |
| Online leisure engagement | 0.762 | 0.223 | 1.000 | 0.357 | 0.116 | 0.282 |
| Learning engagement | 0.368 | 0.370 | 0.357 | 1.000 | 0.154 | 0.355 |
| Social engagement | 0.143 | 0.098 | 0.116 | 0.154 | 1.000 | 0.335 |
| Friend gathering | 0.299 | 0.256 | 0.282 | 0.355 | 0.335 | 1.000 |

Panel B. Variance inflation factors

| **Variable** | **VIF** | **N used for VIF** |
| --- | --- | --- |
| Internet media use frequency | 3.375 | 5551 |
| Online leisure engagement | 3.284 | 5551 |
| Learning engagement | 1.214 | 5551 |
| Social engagement | 1.123 | 5551 |
| Friend gathering | 1.245 | 5551 |

**Note:** Panel A reports Spearman correlations because the focal and lifestyle-overlap variables are ordinal. Panel B reports VIF values for internet media-use frequency and the four additional lifestyle-overlap covariates. Correlations and VIF values are rounded to three decimal places.

**Supplementary Table S12. Ordered logit marginal effects**

| **Physical activity category** | **Predicted probability at observed internet use** | **Predicted probability after +1 level internet use** | **Probability difference** | **Average marginal effect for +1 level internet use** |
| --- | --- | --- | --- | --- |
| 1 | 0.422 | 0.404 | -0.018 | -0.018 |
| 2 | 0.130 | 0.131 | 0.001 | 0.001 |
| 3 | 0.112 | 0.114 | 0.002 | 0.002 |
| 4 | 0.134 | 0.138 | 0.004 | 0.004 |
| 5 | 0.203 | 0.213 | 0.011 | 0.011 |

**Note:** The ordered logit model treats the five-level physical activity participation frequency variable as ordinal. Predicted probabilities and average marginal effects are rounded to three decimal places and should be interpreted descriptively.

**Supplementary Table S13. Ordered logit health robustness**

| **Health-related outcome** | **Predictor** | **Ordered logit coefficient** | **SE** | **95% CI** | **p value** | **N** | **Log likelihood** | **Province FE** |
| --- | --- | --- | --- | --- | --- | --- | --- | --- |
| Self-rated health | Internet media-use frequency | 0.138 | 0.021 | [0.098, 0.178] | <0.001 | 5621 | -7891.361 | No |
| Self-rated health | Physical activity participation frequency | 0.118 | 0.016 | [0.086, 0.150] | <0.001 | 5621 | -7891.361 | No |
| Mental wellbeing | Internet media-use frequency | 0.111 | 0.021 | [0.070, 0.152] | <0.001 | 5613 | -7680.810 | No |
| Mental wellbeing | Physical activity participation frequency | 0.116 | 0.017 | [0.083, 0.148] | <0.001 | 5613 | -7680.810 | No |
| Daily functioning | Internet media-use frequency | 0.130 | 0.021 | [0.090, 0.170] | <0.001 | 5616 | -7783.125 | No |
| Daily functioning | Physical activity participation frequency | 0.141 | 0.017 | [0.109, 0.174] | <0.001 | 5616 | -7783.125 | No |

**Note:** Ordered logit models treat self-rated health, mental wellbeing and daily functioning as five-level ordinal outcomes. Models include internet media-use frequency, physical activity participation frequency, age, age squared, gender, years of education, log income, rural residence, agricultural hukou and marital status. Province fixed effects were omitted from these ordered models to avoid computational instability in high-dimensional ordinal specifications.

Supplementary Figures

**Supplementary Figure S1. Physical activity distribution.**


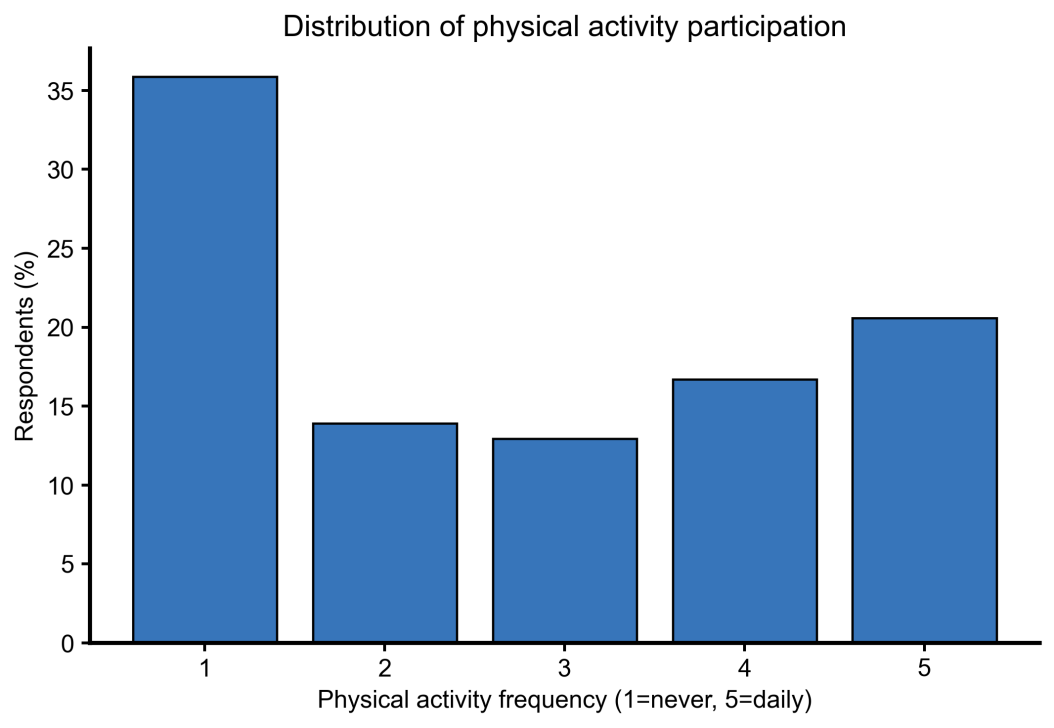


**Supplementary Figure S2. Residence-specific descriptive pattern.**


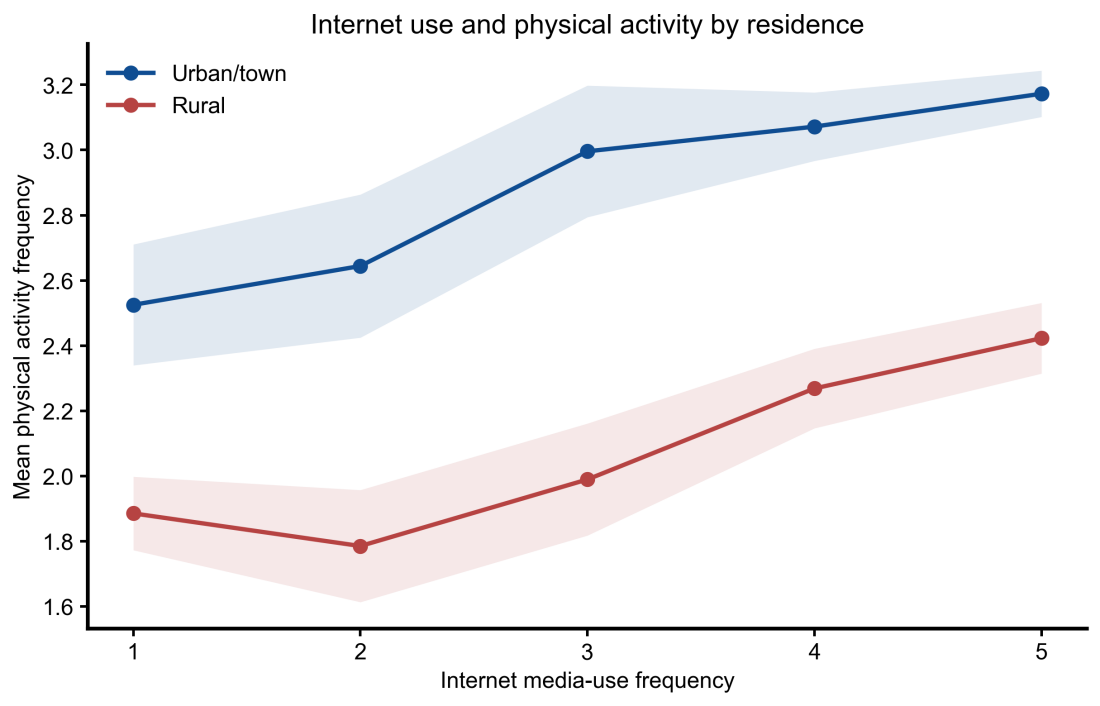


**Supplementary Figure S3. Subgroup heterogeneity.**


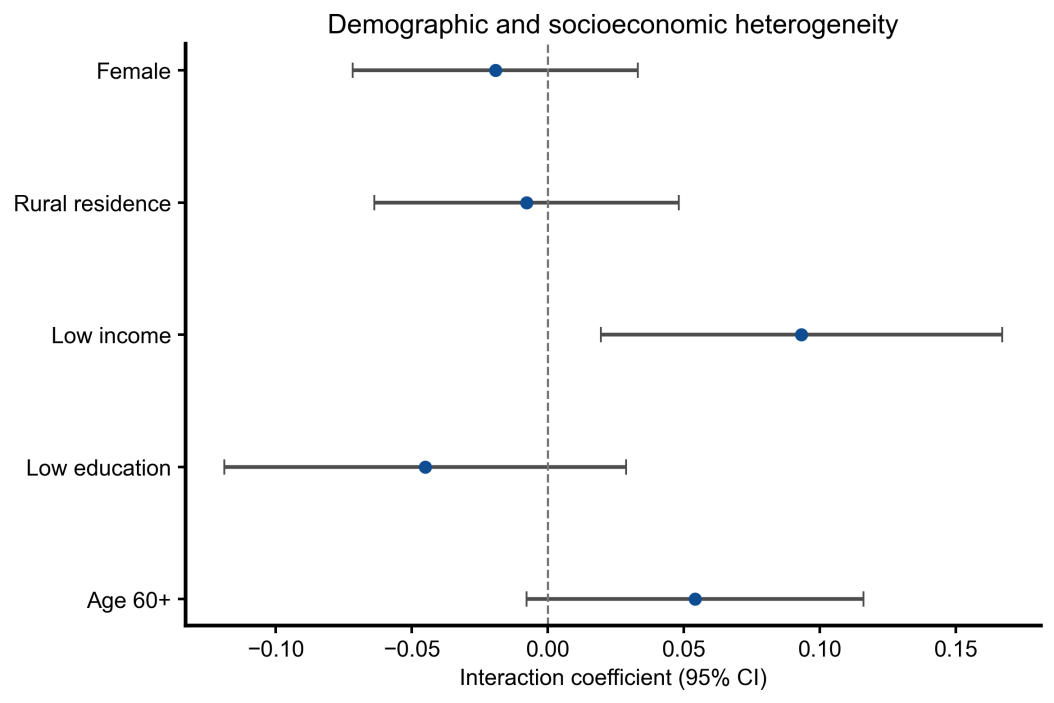


**Supplementary Figure S4. Lifestyle-overlap attenuation.**


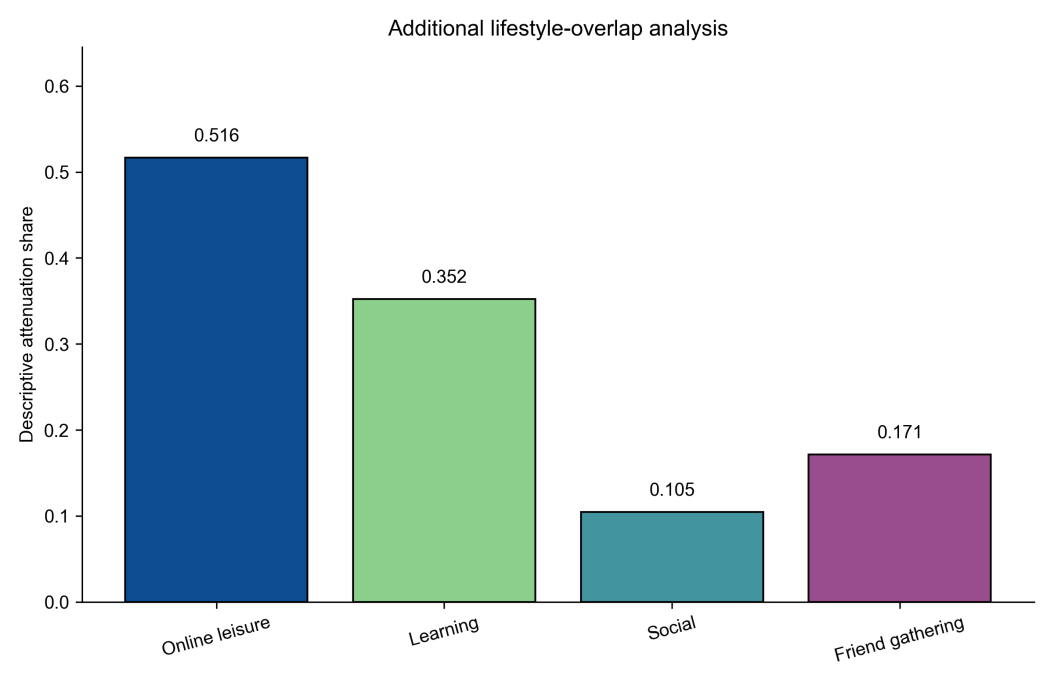

Supplement: Supplementary file 1 [file Table_1.docx]
